# Supplementary material for: Sensitive dissection of a genomic regulatory landscape using bulk and targeted single-cell activation
Source: Cell Genom. 2025 Sep 9;5(10):100984. doi: 10.1016/j.xgen.2025.100984 (PMC12790989; doi:10.1016/j.xgen.2025.100984)
Supplement: Document S1. Figures S1–S7 and Tables S1 and S2 [file mmc1.pdf]

Cell Genomics, Volume *5*

Supplemental information

Sensitive dissection of a genomic regulatory landscape using bulk and targeted single-cell activation

Dubravka Vučićević, Che-Wei Hsu, Lorena Sofia Lopez Zepeda, Martin Burkert, Antje Hirsekorn, Ilija Bilić, Nicolai Kastelić, Markus Landthaler, Scott Allen Lacadie, and Uwe Ohler

Figure S1

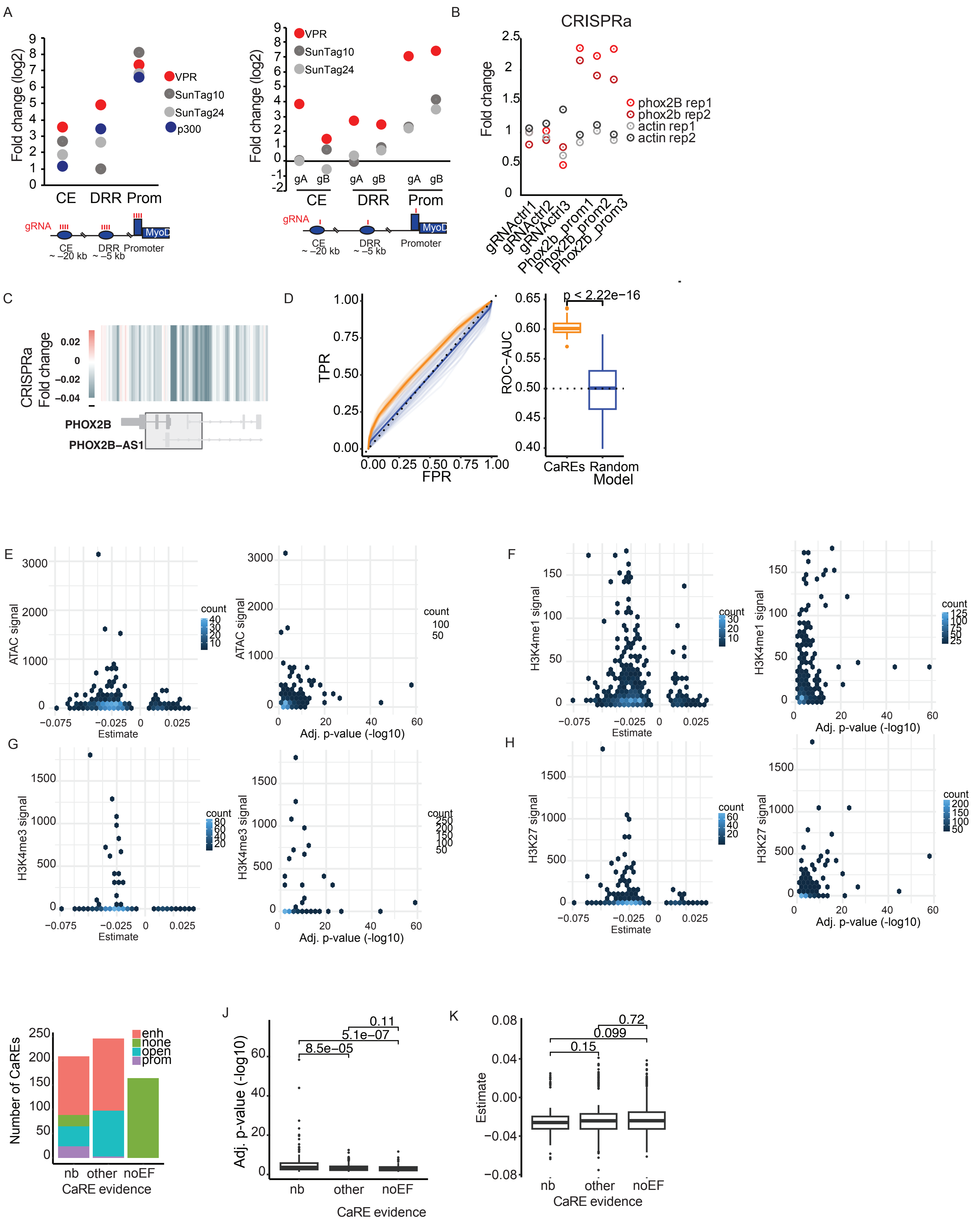

A
Fold change (log2)
VPR
SunTag10
SunTag24
p300
CE DRR Prom
gRNA
MyoD
CE ~ −20 kb
DRR ~ −5 kb
Promoter
gA gB gA gB gA gB
B
CRISPRa
Fold change
phox2B rep1
phox2b rep2
actin rep1
actin rep2
gRNActrl1
gRNActrl2
gRNActrl3
Phox2b_prom1
Phox2b_prom2
Phox2b_prom3
C
CRISPRa Fold change
PHOX2B
PHOX2B−AS1
D
TPR
FPR
p < 2.22e−16
ROC−AUC
CaREs
Random Model
E
ATAC signal
Estimate
count
Adj. p-value (-log10)
F
H3K4me1 signal
G
H3K4me3 signal
H
H3K27 signal
I
Number of CaREs
enh
none
open
prom
nb other noEF
CaRE evidence
J
Adj. p-value (-log10)
8.5e−05
5.1e−07
0.11
K
Estimate
0.15
0.099
0.72

Figure S2

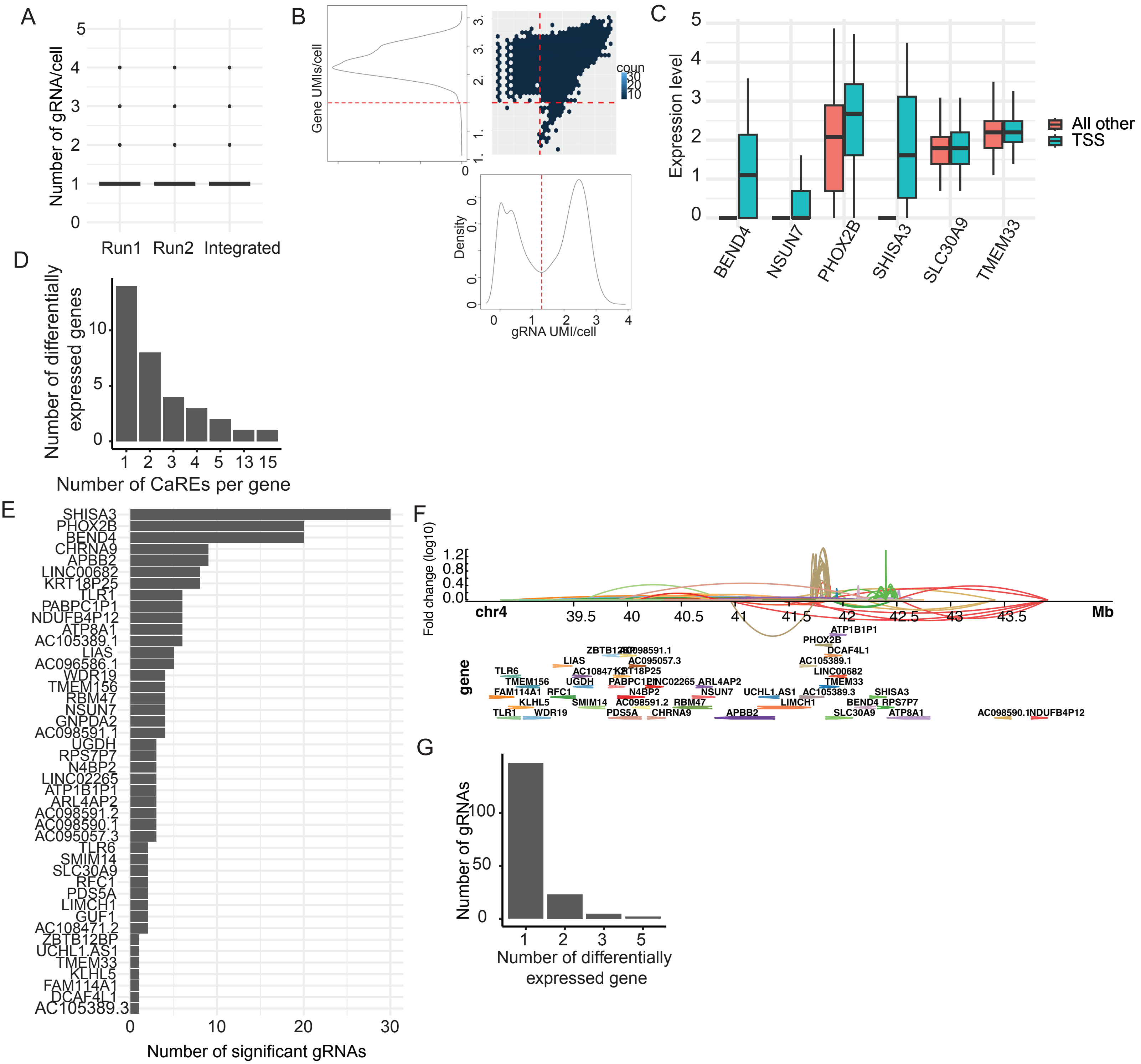
A
Number of gRNA/cell
Run1
Run2
Integrated
B
Gene UMIs/cell
Density
gRNA UMI/cell
count
C
Expression level
All other
TSS
BEND4
NSUN7
PHOX2B
SHISA3
SLC30A9
TMEM33
D
Number of differentially expressed genes
Number of CaREs per gene
E
Number of significant gRNAs
SHISA3
PHOX2B
BEND4
CHRNA9
APBB2
LINC00682
KRT18P25
TLR1
PABPC1P1
NDUFB4P12
ATP8A1
AC105389.1
LIAS
AC096586.1
WDR19
TMEM156
RBM47
NSUN7
GNPDA2
AC098591.1
UGDH
RPS7P7
N4BP2
LINC02265
ATP1B1P1
ARL4AP2
AC098591.2
AC098590.1
AC095057.3
TLR6
SMIM14
SLC30A9
RFC1
PDS5A
LIMCH1
GUF1
AC108471.2
ZBTB12BP
UCHL1.AS1
TMEM33
KLHL5
FAM114A1
DCAF4L1
AC105389.3
F
Fold change (log10)
chr4
Mb
gene
G
Number of gRNAs
Number of differentially expressed gene

Figure S3

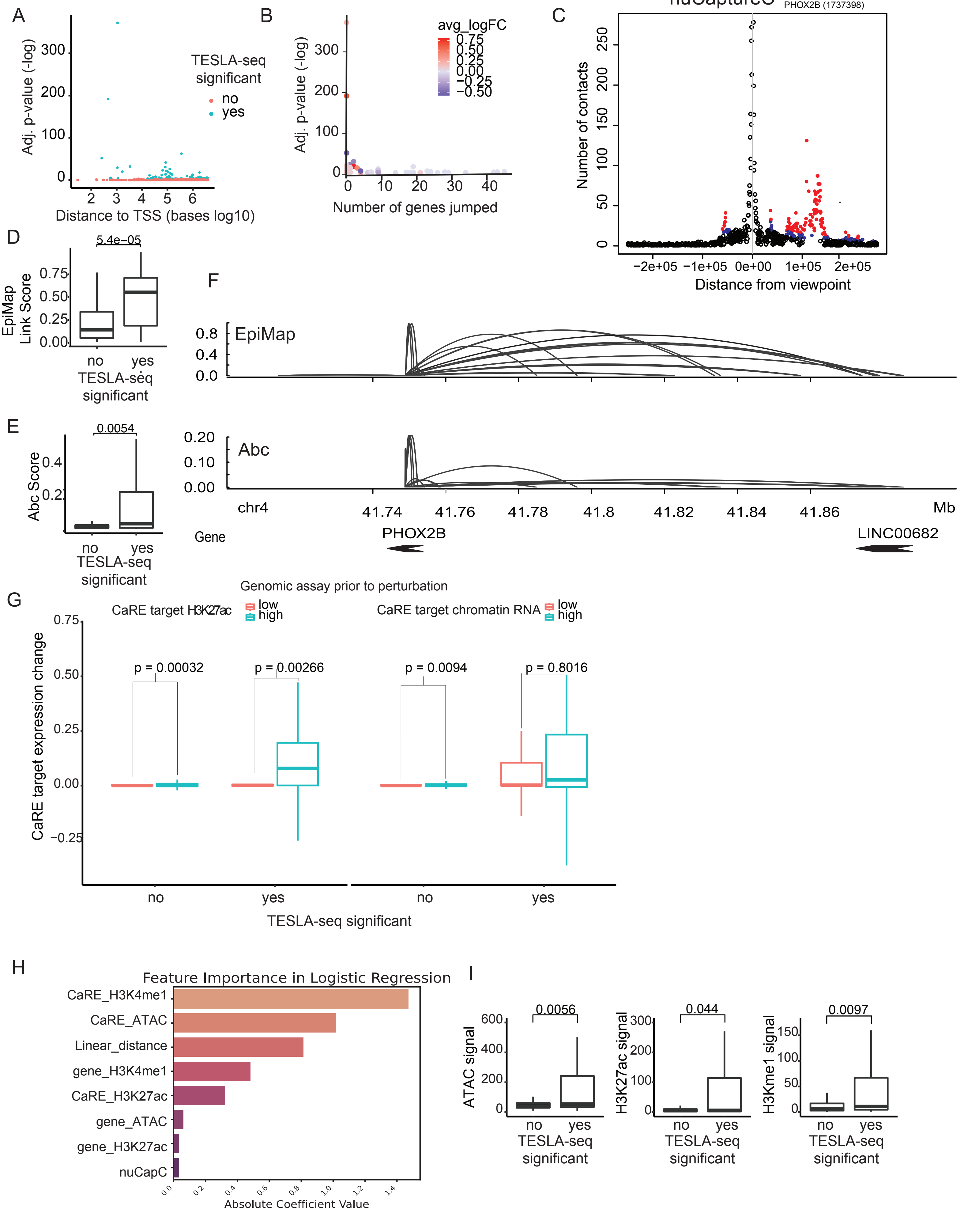

A
Adj. p-value (-log)
Distance to TSS (bases log10)
TESLA-seq significant
no
yes
B
avg_logFC
Adj. p-value (-log)
Number of genes jumped
C
nuCaptureC
PHOX2B (1737398)
Number of contacts
Distance from viewpoint
D
EpiMap Link Score
5.4e-05
TESLA-seq significant
E
Abc Score
0.0054
TESLA-seq significant
F
EpiMap
Abc
chr4
Mb
Gene
PHOX2B
LINC00682
G
Genomic assay prior to perturbation
CaRE target H3K27ac
CaRE target chromatin RNA
low
high
CaRE target expression change
p = 0.00032
p = 0.00266
p = 0.0094
p = 0.8016
TESLA-seq significant
H
Feature Importance in Logistic Regression
CaRE_H3K4me1
CaRE_ATAC
Linear_distance
gene_H3K4me1
CaRE_H3K27ac
gene_ATAC
gene_H3K27ac
nuCapC
Absolute Coefficient Value
I
ATAC signal
0.0056
H3K27ac signal
0.044
H3Kme1 signal
0.0097
TESLA-seq significant

Figure S4

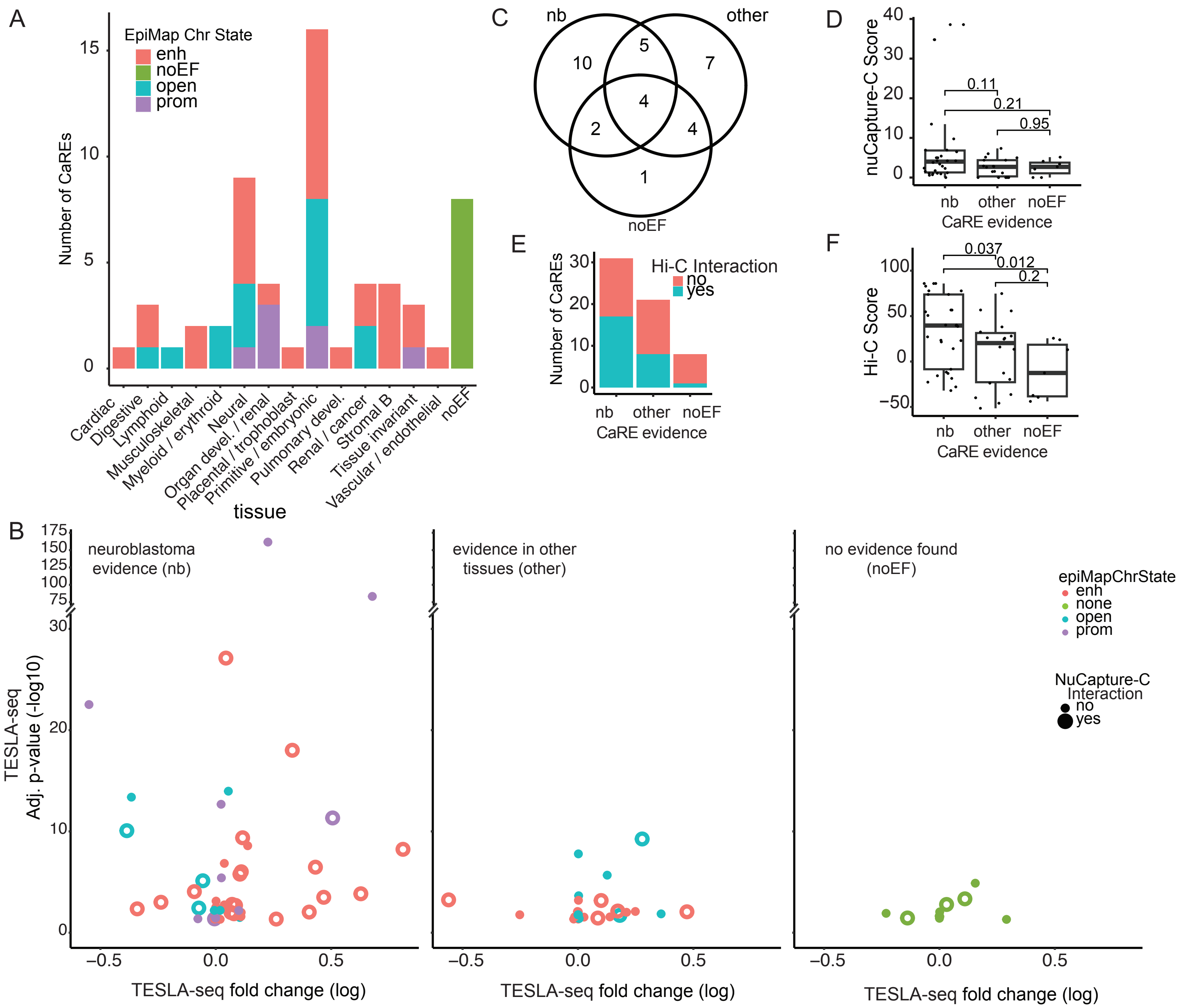
A
EpiMap Chr State
enh
noEF
open
prom
Number of CaREs
tissue
Cardiac
Digestive
Lymphoid
Musculoskeletal
Myeloid / erythroid
Neural
Organ devel. / renal
Placental / trophoblast
Primitive / embryonic
Pulmonary devel.
Renal / cancer
Stromal B
Tissue invariant
Vascular / endothelial
noEF
C
nb
other
noEF
10
5
7
4
2
4
1
D
nuCapture-C Score
0.11
0.21
0.95
nb
other
noEF
CaRE evidence
E
Number of CaREs
Hi-C Interaction
no
yes
nb
other
noEF
CaRE evidence
F
Hi-C Score
0.037
0.012
0.2
nb
other
noEF
CaRE evidence
B
neuroblastoma evidence (nb)
evidence in other tissues (other)
no evidence found (noEF)
TESLA-seq Adj. p-value (-log10)
TESLA-seq fold change (log)
epiMapChrState
enh
none
open
prom
NuCapture-C Interaction
no
yes

Figure S5

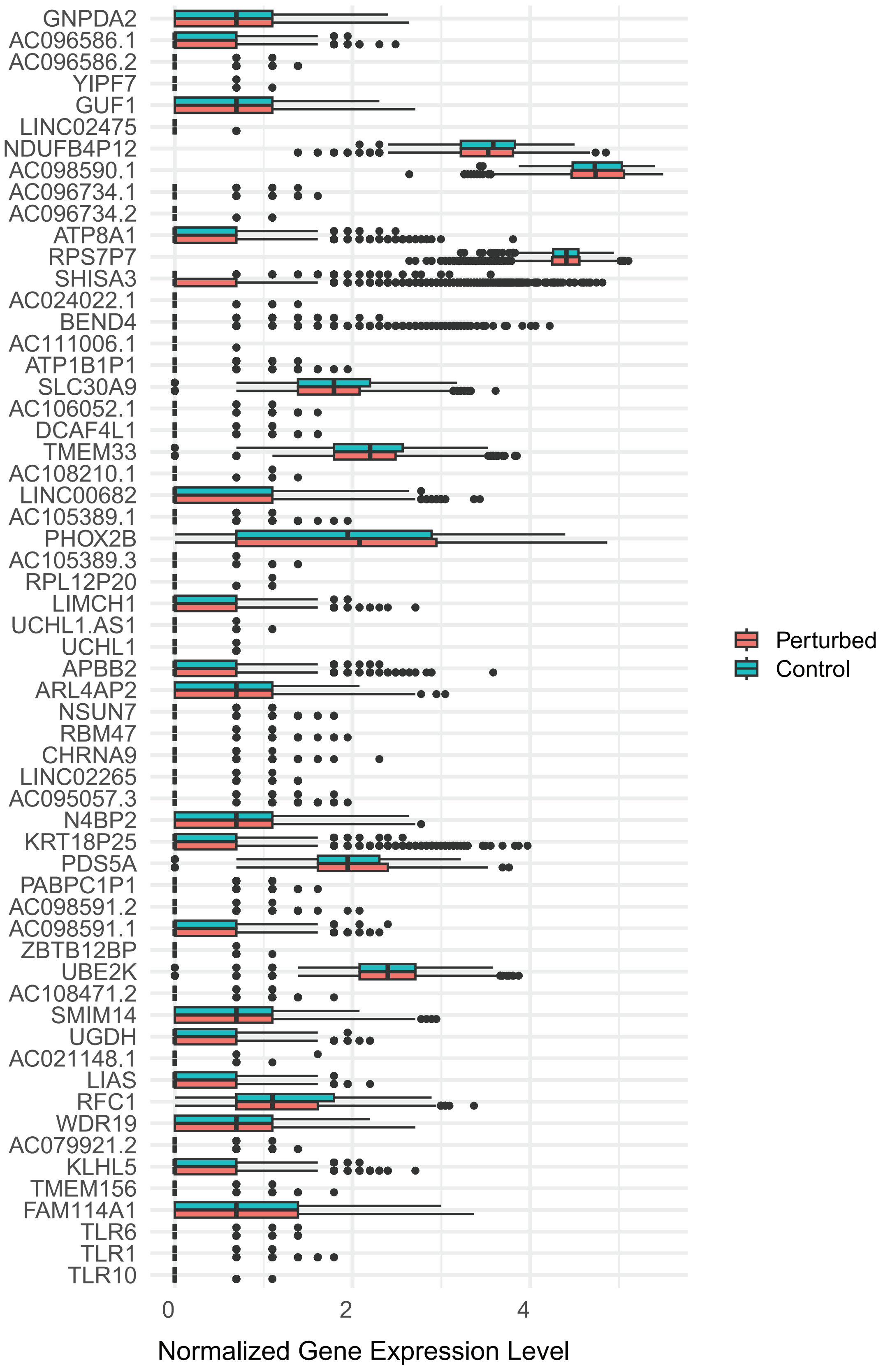
GNPDA2
AC096586.1
AC096586.2
YIPF7
GUF1
LINC02475
NDUFB4P12
AC098590.1
AC096734.1
AC096734.2
ATP8A1
RPS7P7
SHISA3
AC024022.1
BEND4
AC111006.1
ATP1B1P1
SLC30A9
AC106052.1
DCAF4L1
TMEM33
AC108210.1
LINC00682
AC105389.1
PHOX2B
AC105389.3
RPL12P20
LIMCH1
UCHL1.AS1
UCHL1
APBB2
ARL4AP2
NSUN7
RBM47
CHRNA9
LINC02265
AC095057.3
N4BP2
KRT18P25
PDS5A
PABPC1P1
AC098591.2
AC098591.1
ZBTB12BP
UBE2K
AC108471.2
SMIM14
UGDH
AC021148.1
LIAS
RFC1
WDR19
AC079921.2
KLHL5
TMEM156
FAM114A1
TLR6
TLR1
TLR10
0
2
4
Normalized Gene Expression Level
Perturbed
Control

Figure S6

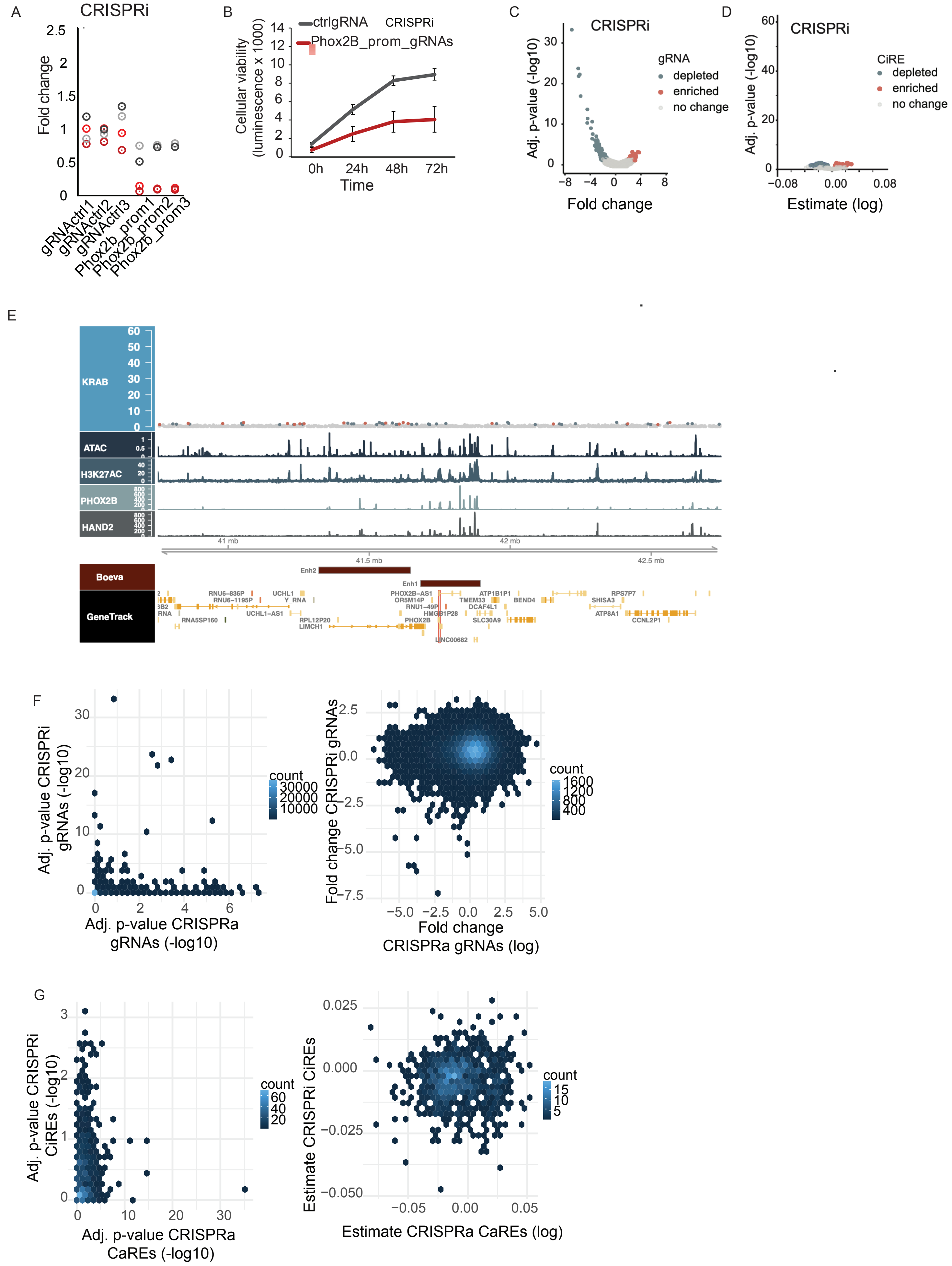

A
CRISPRi
Fold change
gRNActrl1
gRNActrl2
gRNActrl3
Phox2b_prom1
Phox2b_prom2
Phox2b_prom3
B
ctrlgRNA
CRISPRi
Phox2B_prom_gRNAs
Cellular viability (luminescence x 1000)
0h
24h
48h
72h
Time
C
CRISPRi
gRNA
depleted
enriched
no change
Adj. p-value (-log10)
Fold change
D
CRISPRi
CiRE
depleted
enriched
no change
Adj. p-value (-log10)
Estimate (log)
E
KRAB
ATAC
H3K27AC
PHOX2B
HAND2
41 mb
42 mb
41.5 mb
42.5 mb
Boeva
Enh2
Enh1
GeneTrack
F
Adj. p-value CRISPRi gRNAs (-log10)
Adj. p-value CRISPRa gRNAs (-log10)
count
Fold change CRISPRi gRNAs
Fold change CRISPRa gRNAs (log)
G
Adj. p-value CRISPRi CiREs (-log10)
Adj. p-value CRISPRa CaREs (-log10)
Estimate CRISPRi CiREs
Estimate CRISPRa CaREs (log)

Figure S7

A

CaRE	Closest CaRE	distance (bp)	CaRE_target	closestCaRE_target
CaRE_1	CaRE_3	3671	RBM47,NSUN7,RPS7P7	PDS5A,TLR1
CaRE_171	CaRE_174	9631	NDUFB4P12	APBB2
CaRE_174	CaRE_179	9129	APBB2	TLR1
CaRE_553	CaRE_557	4752	PHOX2B,LINC00682	PHOX2B
CaRE_561	CaRE_562	1557	PHOX2B	PHOX2B
CaRE_668	CaRE_669	1133	CHRNA9	BEND4
CaRE_671	CaRE_669	2254	BEND4,AC098590.1	BEND4
CaRE_729	CaRE_730	1020	SHISA3,ZBTB12BP	BEND4,SHISA3
CaRE_740	CaRE_741	826	SHISA3,BEND4	SHISA3
CaRE_789	CaRE_790	654	SHISA3	SHISA3,TLR6,UGDH
CaRE_803	CaRE_806	5134	SHISA3	SHISA3,TMEM156,PABPC1P1
CaRE_828	CaRE_829	1266	SHISA3,TLR6,LINC02265	SHISA3

B

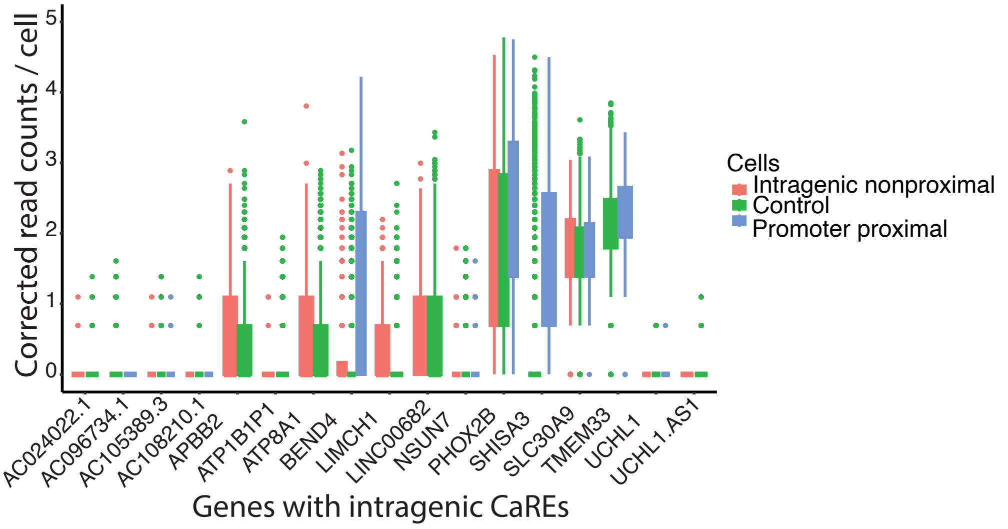

C

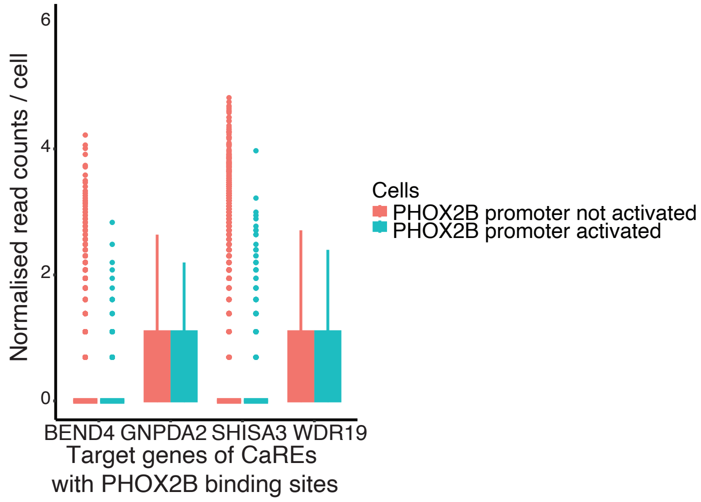

Supplementary Figure legends:

Figure S1. Details of the bulk CRISPR activation screen related to Figure 1. A) Upregulation of MyoD in HEK293 cells via different indicated CRISPRa constructs at three different non-coding loci via pools of four gRNAs (left) or individual gRNAs (right). Relative expression of target genes is determined by RT- qPCR and normalized to GAPDH. Shown are fold changes relative to the control at a log2 scale. **B)** PHOX2B expression changes upon activation with dCas9-VPR with control or gRNAs targeting the promoter of Phox2B in SH-SY5Y cell line. Relative expression of target genes is determined by RT- qPCR and normalised to GAPDH. Shown are fold changes relative to the control. Expression of actin is shown as a control. **C)** CaREs signal at the PHOX2B promoter. Significantly enriched/depleted CaREs (FDR<0.05) are shown in red and blue respectively. **D)** Evaluation of epigenetic model for CaREs prediction. Left panel: Receiver operator characteristic (ROC) of trained CaRE models (light orange, n=50) and random models (light blue, n=50) from 10 repeats of a 5-fold cross validation each. Mean ROC curve for CaRE and random models in dark orange and blue respectively. TPR: True positive rate, FPR: False positive rate. Right panel: Distributions of ROC area under the curve (ROC-AUC) for CaRE and random models. Boxplot midlines mark median; upper and lower hinges extend to first and third quartile; upper and lower whiskers extend to the smallest and largest value max. 1.5 × IQR. Comparison p-value from two-sided Wilcoxon ranksum test. **E-H)** The association of CaREs to open chromatin - ATAC (E), H3K4me1 signal (F), H3K4me3 signal (G) and H3K27ac signal (H) relative to the CaRE fold change (estimate, left) or adjusted p-value (right). **I)** Number of CaREs identified in the phenotypic screen in each category, nb, other, noEF grouped by the EpiMap classification indicated with colours. **J-K)** Comparison between different CaRE evidence categories (nb, other, noEF) in CaRE-gene pair adj. P-value (J) and fold change (estimate, K). All indicated p-values were calculated using the two-sided Wilcoxon Rank Sum test.

Figure S2. Details of the TESLA-seq related to Figure 2. A) Number of gRNA detected per cell in each TESLA-seq run and both together (integrated). **B)** Cell density plot. The red line represents thresholds used for cell filtering. Cells having either gene UMI count per cell or gRNA UMI counts per cell below thresholds are filtered. **C)** TESLA-seq normalized gene expression level, comparing cells having gRNA targeting indicated gene promoter versus all other cells. **D)** Histogram showing the number of CaRE that induce differential expression of an indicated number of genes. **E)** Number of gRNA that significantly cause differential gene expression of an indicated gene. **F)**. Genome browser snapshot representing genomic distance and regulatory relationship between a gRNA and a gene determined by TESLA-seq. Each link represents an effect of a gRNA on the gene. Color reflects the target gene. **G)** Histogram showing the number of genes that are differentially expressed due to perturbation of an indicated number of gRNA.

Figure S3. Additional details of properties of CaREs and their targets identified by TESLA-seq related to Figure 3. A) Relationship between adjusted p-values (-log10) of each CaRE-gene regulatory pair and genomic distance between the CaRE and the gene's transcriptional start site (in bases at log10 scale). **B)** Relationship between adjusted p-values (-log10) of each CaRE-gene regulatory pair and number of genes located between the CaRE and the gene (jumped genes). Color depicts the average fold change. **C)** Number of contacts detected for PHOX2B by nuCaptureC centered on the PHOX2B TSS (red: highly significant interactions (pLevel = 5, blue: significant interactions, pLevel = 3, black: background interactions**). D)** Comparison of EpiMap link score between TESLA-seq significant and non-significant CaRE-gene regulatory pairs**. E)** Comparison of Abc score between significant and TESLA-seq non-significant CaRE-gene regulation pairs**. F)** Genome browser snapshot of PHOX2B regulatory regions determined by EpiMap (top) and Abc (bottom). **G)** Comparison of the CaRE target gene expression (average fold change at log 2 scale) between TESLA-seq significant and non-significant CaRE-gene regulation pairs that have a low or high: H3K27 signal at the promoter of the target gene (left) and chromatin RNA signal (right) (non-significant). **H)** CaRE features that contribute the most to the predictability of a logistic regression model. **I)** Comparison of ATAC signal (left), H3K27ac signal (middle) and H3K4me1 signal (right) between significant and non-significant CaREs**.** All indicated p-values were calculated using the two-sided Wilcoxon Rank Sum test.

Figure S4. Additional details of the integration of TESLA-seq results with epigenomic data related to Figure 4. A) Number of CaREs defined by EpiMap in each indicated tissue. The color indicates the EpiMap chromatin state. **B)** Comparison between CaRE evidence categories (nb, other, noEF) relationship between TESLA-seq adjusted p-value (-log10 scale) and fold change (log2 scale). The data is further stratified by: nuCapture-C interaction displayed via circle fill and class defined by EpiMap indicated via circle colour. **C)** Venn diagram displaying an overlap between genes targeted by CaREs in nb, other or noEF classes. **D)** CaRE-gene nuCapture-C score stratified by CaRE evidence class. **E)** Number of CaREs in each evidence categories (nb, other, noEF) grouped by Hi-C detected interaction (yes - 3D interaction is detected or no - no interaction detected). **F)** Comparison between different CaRE evidence categories (nb, other, noEF) in Hi-C score. All indicated p-values were calculated using the two-sided Wilcoxon Rank Sum test.

Figure S5. Normalized expression level of all TESLA-seq captured genes related to Figure 4. Histogram showing normalized gene expression level of all TESLA-seq captured genes in perturbed and control cells.

Figure S6. CRISPRi phenotypic screen related to Supplementary Note 1.
A) PHOX2B expression changes upon repression with dCas9-KRAB with control or gRNAs targeting the promoter of Phox2B in SH-SY5Y cell line. Relative expression of target genes is determined by RT- qPCR and normalised to GAPDH. Shown are fold changes relative to the control.

Expression of actin is shown as a control. **B)** MTT viability assay with control or gRNAs targeting the promoter of Phox2B in a SH-SY5Y-KRAB (CRISPRi) cell line. **C)** Volcano plot showing the log-fold change of gRNA representation between the first and last time-point of the CRISPRi experiment. Significantly enriched/depleted gRNAs (FDR<0.05) are shown in red and blue, respectively. **D)** Volcano plot showing the results for our CRISPRi screen, each dot corresponds to a CaRE defined by the CRISPRa screen. The x axis shows the slope calculated by MLM. Significantly enriched/depleted CaREs (FDR<0.05) are shown in red and blue, respectively. **E)** CiREs signal around the PHOX2B locus (+/- 1 MB). From bottom to top: annotation for PHOX2B and its location within the genome, ChIP-seq signal for HAND2, PHOX2B, H3K27ac, and ATAC-seq signal in SH-SY5Y cell line. At the top is the score and direction (blue for depletion, red for enrichment) of CiREs. *P*-values shown are adjusted for multiple testing (FDR). **F-G)** Comparison between CRISPRa and CRISPRi screen at the gRNA (F) and CaRE/CiRE level (G) in adjusted p-value (left) or fold change (estimate, right).

Figure S7. Characteristics of CRISPR activation related to Supplementary Note 2. A) Table showing the closest significant CaRE neighbors and their target genes identified by TESLA-seq. **B)** Genes expression comparison in cells in which the following CaREs are targeted: CaREs targeting the promoter of the gene depicted (promoter proximal), CaREs within genes that are not at the promoters of genes (intragenic nonproximal) and CaREs that are targeting genes other than the one depicted (control).**C)** Genes expression comparison of the genes targeted by CaREs that have PHOX2B binding sites in cells in which the PHOX2B promoter was activated (blue) and cells in which PHOX2B was not activated (red).

name	sequence
phox2b_qPCR_FW	GGAGACTCACTACCCCGACA
phox2b_qPCR_RV	CTCCTGCTTGCGAAACTTG
myoD_qFW	AGCACTACAGCGGCGACT
myoD_qRV	GCGCCTTCGTAGCAGTTC
APBB2_qFW	TGCTGGTAACGTGTCTGAGG
APBB2_qRV	GGAGGTGGTCGAACTTTCTG
SHISA3_qFW	GAGCACCCAGGCATCACT
SHISA3_qRV	AACAGGTGCAACAATAAATAGCC
actin_FW	CGACAGGATGCAGAAGGAG
actin_RV	GTACTTGCGCTCAGGAGGAG
gapdh_FW	GCTCTCTGCTCCTCCTGTTC
gapdh_RV	ACGACCAAATCCGTTGACTC
phox2b_prom1REVgRNA	aaactgccctttaattcaatcacaC
phox2b_prom1FWDgRNA	CACCGtgtgattgaattaaagggca
phox2b_prom2REVgRNA	aaacccccttctaaccagctccctgC
phox2b_prom2FWDgRNA	CACCGcagggagctggttagaaggg
phox2b_prom3REVgRNA	aaacctgatcctcccttctaaccaC
phox2b_prom3FWDgRNA	CACCGtggttagaagggaggatcag
phox2b_prom4REVgRNA	aaacCCCTATCATTGATTCCTGCAC
phox2b_prom4FWDgRNA	CACCGTGCAGGAATCAATGATAGGG
phox2b_prom5FWDgRNA	CACCGGAATCAATGATAGGGAGGT
phox2b_prom5REVgRNA	aaacACCTCCCTATCATTGATTCC
phox2b_prom6FWDgRNA	CACCGCGTCTATTGGGCTGGCACTG
phox2b_prom6REVgRNA	aaacCAGTGCCAGCCCAATAGACGC
Neg_ctrl_gRNA_FWgRNA	caccGTATTACTGATATTGGTGGG
Neg_ctrl_gRNA_RVgRNA	aaacCCCACCAATATCAGTAATAC

Table S1. Primers and individual gRNAs oligoes, related to Figure 1.

name	sequence
HIC_chr4_91471	GATCAATAGCCCCAAGGTCACTAATCATTAATCTAGACAAAATAGTAAATACTAAAAATCTAAATAACCC
HIC_chr4_91683	GATCTCTTTTTCAGAAGAAGAGAAAATAACTTGTAAATGAACGGAAATACAATTGAAAATGCTTGACAAA
HIC_chr4_91683	TACTGGTTGATACATATTTATGACTACATATTTAGCCATATGTACGAGCAATCATACCCACATTCTGATC
HIC_chr4_91712	GATCTGGCGAACCCCGAGACCCACCCGCCCTGGGCTGGAGAGGCGGCGCGCTCTGGCTTTCCGCGCTGGC
HIC_chr4_91712	ACACCCACACGCCCACACTCAGGGTCTGCCCCCTCGGCCTGCGTGAACCTCCGCGGAGCCTGCCTGGATC
HIC_chr4_92179	GGTGAATGTCAGTCATGTCTAGGTTGCATGCACATGACTCATTCATTTACTTATTAAATGATTGTCGATC
HIC_chr4_92220	GATCATAATTTCGATGCCACAAACCCACCACCTAGAGCACACTAATTATCAGATTATTGCAAGGGGAATG
HIC_chr4_92220	TTCCCGAGACCCGGCGTGCCTGGTGAAACTTTTGCATTTAGGCATTTATTTCAGTGCATGCTCTGGGATC
HIC_chr4_92630	GATCCAAACTCTAAAGGAAAACTATTGGAGCTCATTTTTCCTGCCCACGTTAGCTAGCACATTTTGTTGA
HIC_chr4_92630	GACTGCAGACGCCGTAGAAGCGGTGCAGAAAGTGGGAACCCCTCCCTGGCCGAAATGAGCGGACTGGATC
HIC_chr4_93108	GATCCATTCGCGCCAACAACTTCTCCCGCGAAGTGCAAGAAGGCGAAGACAGTGGCGCGCGGTGATGACG
HIC_chr4_93108	GACATCGAGAGGAACCACGGTGCTTTGGTGTGAGCTCGGTTTTTGGCGGGGGCGCTAAAGTAGGGGGATC
HIC_chr4_93406	GATCATGTTCTGTACATGACTACAAATAGTCCGAACGGTAGCCAGTTCCTTTCTGTTACCCCACCATTTG
HIC_chr4_93406	AGCAGCTTGGCTGCTTGTCATAAATGGAGCGACGTAATTTCGACCTGTCCTTTCCCGGGAGTTAGCGATC
HIC_chr4_93611	GATCTGCGGGTCGGTCTCGGCGGGCGGATTACTCCTCGCCCACAACCTCGAGGCCCCGGGGTCAACCGCG
HIC_chr4_93611	AAGAAAGTGATTAGTAATTTATAAAGGCACATTAAAATACCATATAAATTACGGTTTTCTTGATAGGATC
HIC_chr4_93953	GATCTGCCGGCTCCCGCGGGCGGCGGCGCAACAGATTGCAGCGCCTGGAGACTCCAGCTGCCCCGCCTGC
HIC_chr4_94155	GATCCGGGAGAGCGTTTTCCTGCGCTAGACACGGCGTTCAGCCTCCGGGTTCCGGGTCTAGCTGAGTCAG
HIC_chr4_94155	GAGGGAGCAGGCCGAGCCTGAGAAAACCCGGGAAGTGGGTTGGGGGAAGGGGAAAGGTGGTAACTGGATC
HIC_chr4_94380	GTAGCTCTGGGATAGAGAAAACTTCCCAAGGGATGCTGATGCTGCTGGTTGGATGAACAACACTGAGATC
HIC_chr4_95059	GATCAATTTTAAACAGCAGGAACACCAATGGCACTGTTAACTGCTTTCTGGGTAGCCTCTTTAGCTTGGT
HIC_chr4_95073	GATCGAACCCCGCCGGCCCCGCCAACCCGGCCCTGCCAGCCCCAGGCTCACGGGGCTCCTCCGCACCAGC
HIC_chr4_95073	AGACGAAACTGGTTGGGAGGTAGTTAATTGCTCGGTGAAAATGAACTGATTTCCTCCTCAGGGAGAGATC
HIC_chr4_95258	CTACGGCGCTCGGCCAGTCAGCAGCTCTGCCAGCATCTATGCAGGTGCCGGGGGCTCTGGTTCCTGGATC
HIC_chr4_95387	GATCCACCTGGGCGACGTGGCAGCCATTGCCGGCGCCTCGAGACCCCTCCCCACCCGGCCGCCCACCCGC
HIC_chr4_95387	AGCGGCGGGCGGCGCGGCGAATGAACCCCCAAGCCCTGAATGTGGGGCCCGGCGCGGGCCGCCTCCGATC
HIC_chr4_96122	GATCATTGTGACATTATAATGGTGAAAAATTAGAAATAACCTAAACATCTAGCATTTAGAGAATGATTAA
HIC_chr4_96122	GCTTTGCCACCCAGTTTTGGTCATGGGGACTTGAGTTACTGTGACCTCAGAGGCCCCTAGGGGCCTGATC
HIC_chr4_96184	GATCTCGGTCCTGCATGCAATGCAAGCCTGAGCTCTCCCGCCATAAGGCTGCAGCGGTGTGGGCTCCTTG
HIC_chr4_96184	CTGCATGCAATGCAAGCCTGAGCTCTCCCGCCATAAGGCTGCAGCGGTGTGGGCTCCTTGTGCCCAGATC

HIC_chr4_97125	GATCTCTGCGACCCCCGCGTGCCCGGGGAGAGCTCCCGGGGCGAGCTCCCGGGGCGTCCTTACCTGGGGC
HIC_chr4_97125	TAATCCCTTTCCATTAGTGAGGCCCTTTCTACTTTATGGAGCACTTTCACCCTTCTTACTTTATGTGATC
HIC_chr4_97617	GATCAGTTACCAGGAGAAGTTCTAAAGCAAGAAGAGAAAAGCATTTCAATTTGGGACATTTATTTGCACC
HIC_chr4_97617	TTTGCAGCCTACCTGTGCAATCATAGGAGATGGCCTAAAGGAAGGACTTGAGAAACTACATGATATGATC
HIC_chr4_98797	GATCCCCGGAACCCGGCCTGGCCACCCCGCTCTCTCTCGGGGGTCCGGGGTAGGTGAGCGGAGCCTGCCC
HIC_chr4_98797	ACGGCCACCAAGCGTTGCGCAGCTGCAGGAGGAAATTCCCTTAATTATGAATTTACAGAGGGGACTGATC
HIC_chr4_98914	GATCTGTTTTCTCAAGTCTCCAATCGCCTGCCTTCTTTGTGTCTTGTATTACCCTCACATCCCCCAGCTT
HIC_chr4_98914	TGTTTTTCGTCTTCCCTAGGCTATTTCTGCCGGGCGCTCCGCGAAGATGCAGCTCAAGCCGATGGAGATC
HIC_chr4_99200	GATCGTCACTTGGGCTGTAGTGCAAAACAGAAGGCATGCTCAAGTGGGAGTGGCCAAGGAGAGTTTAAAG
HIC_chr4_99200	AGAAGGCATGCTCAAGTGGGAGTGGCCAAGGAGAGTTTAAAGGAAGGGTTAGAAAGAAGTGAGCCGGATC
HIC_chr4_99266	ATCTCATATCCTGGTTCCTCTGACACCAGCTGCCTCTCCCATACCACCTAAGTTTGACCCCAGTGAGATC
HIC_chr4_100160	GATCCCGGCGTGAGGGAAGGGCAGCCGGACGTGGCCCCAAAAGTGGTCCTTATCGGGTTATACTGGAAGC
HIC_chr4_100160	TCCCTATCATTGATTCCTGCATCTCTAATTAGAATTTAATACCACACCATTACGCACCGAGCCCCTGATC
HIC_chr4_100475	GATCCGTTTTCAAACGGCGCGGGGACGGCAGTGCCGGAGGCCGCGTCTCCTTAGTAATCGCGCGGGCAGG
HIC_chr4_100475	CGGATAGGCCGGGCTGCCGCCAAACAAAGAGATAATAAAAAATTAACTATTTTAACATATATTACAGATC
HIC_chr4_100617	GATCATTTGTGAGCTGTATTTAATGCAAAAGTTGCTCCCCCATCCTGATTTCTTAGCTCACTGGGCCAAT
HIC_chr4_100617	GTTGGCTCTTTAGGGCTTCACCCCGAAGCTCCACCTTCGCTCCCGTCTTTCTGGAAACACCGCTTTGATC
HIC_chr4_100720	TCCCGTTTAGCCAACGAGCTGCGTGTGAGCTGCATGGAGCGGAAAAAGGTCCAAATTCGGAGCTTGGATC
HIC_chr4_100747	GATCAGGCTTGCCTAAAACGAGTTGAAACCAAAGCCATTTTAAGAATCCAAAATATGAGATTAGTTTTGT
HIC_chr4_100747	TTTTCTTACTCTCCCAACTTATTTTCTTAAATTTTCTAAAAGGAAGGAGGGGTGCTACTCACTACGGATC
HIC_chr4_100849	GATCCGCAGTGGCAGTGGTGTGTCCTGTCTGCGGAGAGCCAGGCCAGAGACAATGAGCAACACCTCAGAG
HIC_chr4_100849	GAAGAAATTCTTCTGGAACTCAGAGAAGAAGGAGTTTTTAGGCAGGACTGGTGGCAGTTGGTTTAAGATC
HIC_chr4_101192	GATCTTGGCCCGGGTGGTCGCGCGGTGTTTACGGGGCTTTGGGGTCCTGCTTTCCCCGAGCATCGCGGCC
HIC_chr4_101192	ACTGCAACTGGCGCCATCCGGCGAGGGTCTGGAGGGTGCCCAATTTCAGTAGCCGTTTGAATGAGAGATC
HIC_chr4_101904	GATCGCCACAGGTTGGGACTCTGTGCGGCGCTGGAGTTGGCAGCCCCGGGCGCTATGGCTCCCTTGAGGA
HIC_chr4_101904	GGTCTGTGCGCCAGGGGGACGCGGCCGGGTGGGGGAATCAAGGGATGAGTGGGTGGTCGCGTTTCAGATC
HIC_chr4_102061	GATCTTACTTGAATCCTTCTGAACTAAAATTTGGCAGGTTCATTTATAAAATTTCACATTGAGAAAAAAT
HIC_chr4_102061	AAAAGCCATACAAAAAATAAGCAAAAACATCCTAGGAGCTGTATATGACACAATTCTTGAGGACTTGATC
HIC_chr4_102492	GATCTCCGACACGGTCCTCCGCATGGTGGGCATCGCGGCGGCGGCTGCAGGTGGGTCCTCAGCCCGGACT
HIC_chr4_102492	GAGCCAGGGGGAGCCTCCTTCGCCCCGCCCCGCCCCGCTTCGGACCGTATCACGACTCAAAGGGGGGATC

HIC_chr4_105244	GATCTTAAATGTTATATAATAGAAATATTATATATTTCTAAGGGCCTCAGAATCGTGCAGGCGCAATTGT
HIC_chr4_105244	CGGGTGCAAGCCGAGCGGTTGGCCATAAGAGCCCGGCTGAAACGAGAGTACCTGCTTCAGTACAATGATC
HIC_chr4_105495	GATCTGAAAAAATTCCTGTTGCTTAGTGATGTCTTAATGACCCTGTGTAGGCCCAGGCTAACAAGTGTGT
HIC_chr4_105495	ACTCAAAACCTGCAAATTGCTCAGAATTCAAGAAGTCAGATATTTCTGGAAATAGGAGAGATGGTGGATC
HIC_chr4_106946	GATCCTAAGAAATCCTCCAAATGAAGAAACTTTCAAAGGCTCTGGAAACAATACGAATGATTCTGTCTTC
HIC_chr4_106946	TGATGTCACGACAGCGTGCGGCGTGCAGACGTCGGCAAGCTGCGCCGCCGCTTCGGGTTGCTTCCGGATC
HIC_chr4_106949	GATCCTACGGGGGGTACCTTCGAAAAAAAACGGGCTATGCTGCTGTTGCGTGTGGGTACCCTCTCCTGAC
HIC_chr4_106949	AATTCAAGGTGACTGCCCCCTGGAATCTGATTTAGCCAAGTTTTCAAACGTTGGAGTGCCCCATGCGATC
HIC_chr4_107050	GATCCAAACTATTGTCGCCTTATGCAATTCTCTGGCATCACAAGACAGCATCAAAAAACAACAGAGCACC
HIC_chr4_107050	GCCTGAGGTGAGACGCGAGGAGTTCGGTCCGAGTGCGGTGCGGCTGGGCGCTCTTCTTGCCTGGAAGATC

Table S2. Oligoes used for nuCaptureC, related to Figure 3.
